# Cross-Lagged Associations Between Adolescents’ Depressive Symptoms and Negative Cognitive Style: The Role of Negative Life Events

**DOI:** 10.1007/s10964-015-0308-y

**Published:** 2015-06-03

**Authors:** Karlijn C. M. Kindt, Marloes Kleinjan, Jan M. A. M. Janssens, Ron H. J. Scholte

**Affiliations:** Behavioural Science Institute, Radboud University Nijmegen, P.O. Box 9104, 6500 HE Nijmegen, The Netherlands; Trimbos Institute, P.O. Box 725, 3500 AS Utrecht, The Netherlands; Praktikon, P.O. Box 6909, 6503 GK Nijmegen, The Netherlands

**Keywords:** Depression, Negative cognitive style, Adolescence, Negative life events, Longitudinal

## Abstract

Previous research has established that cognitive theory-based depression prevention programs aiming change in negative cognitive style in early adolescents do not have strong effects in universal settings. Although theories suggest that a negative cognitive style precedes depressive symptoms, empirical findings are mixed. We hypothesized that negative cognitive style may not predict depressive symptoms in adolescents with normative depressive symptoms. Depressive symptoms, negative cognitive style and dependent negative life events were assessed in young adolescents (*N* = 1343; mean age = 13.4 years, *SD* = 0.77; 52.3 % girls) at four time points over an 18-month period. Using a cross-lagged panel design, results revealed that depressive symptoms predicted a negative cognitive style but not vice versa. However, when including dependent negative life events as a variable, depressive symptoms did not prospect a negative cognitive style consistently. When dependent negative life events were used as a time-varying covariate, depressive symptoms and a negative cognitive style were not related. We concluded that negative cognitive style is not predictive of depressive symptoms in a community sample of young adolescents. Moreover, the findings suggest that longitudinal relationships between depressive symptoms and a negative cognitive style are not meaningful when dependent negative life events are not considered.

## Introduction

Depression causes a significant burden to individuals and brings high costs for society (Sobocki et al. 2006) and almost 30 % of all people experience a major depressive episode in their lives (Kessler et al. 2012). As symptoms of depression in adolescence strongly predict depressive episodes in adulthood (Pine et al. 1999), depression rates strongly increase in adolescence (Hankin et al. 1998), and 13 % of the 13- to 17-year olds reported to have experienced a depressive episode (Kessler et al. 2012), depression prevention programs have been developed to decrease the incidence of depression in youth. Most of these prevention programs are based on cognitive theories and teach adolescents skills to change a negative cognitive style into a more realistic, helpful cognitive style (Gillham et al. 2007; Kindt et al. 2014; Pössel et al. 2004). Modification of a maladaptive cognitive style should thus prevent or restrain the increase of depressive symptoms.

Yet, even though research on cognitive theory-based depression prevention programs for adolescents from community samples shows encouraging results, the effects are small and inconsistent across studies (Merry et al. 2012; Stice et al. 2009). This could imply that these prevention programs do not sufficiently impact or change a negative cognitive style in young adolescents. However, another reason for why cognitive theory-based prevention programs might not show the desired effects could be that a negative cognitive style does not have a major impact on depressive symptoms, or that depressive symptoms drive negative cognitive styles and not vice versa. Increased knowledge about the prospective links between a negative cognitive style and depressive symptoms in early adolescence is essential to understand why cognitive theory-based depression prevention programs only show small effects. Therefore, the aim of this study was to examine the longitudinal, bidirectional relationships between a negative cognitive style and depressive symptoms.

Meta-analytic reviews clearly showed that a negative cognitive style and depressive symptoms are cross-sectionally associated in children and adolescents [effect size = .41 in Gladstone and Kaslow (1995); average r = .35, average Z = 4.29, *p*s < .0001 in Joiner and Wagner (1995)]. After childhood, these associations become stronger when youth develop a more stable cognitive style (Cole et al. 2008) and show strong improvements in reasoning and abstract thinking (Steinberg 2005). Previous longitudinal studies have tried to reveal the temporal order of the bidirectional associations between a negative cognitive style and depressive symptoms in adolescents, but so far, findings are mixed. While a previous study showed a negative cognitive style to precede the increase of depressive symptoms, and not vice versa (Kindt et al. 2015), other research showed the opposite effect, namely, that depressive symptoms predicted a negative cognitive style (Hankin et al. 2001; Johnson and Miller 1990; LaGrange et al. 2011; McCarty et al. 2007; Timbremont and Braet 2006). Yet, again other research showed bidirectional effects, with depressive symptoms and a negative cognitive style mutually influencing each other over time (Calvete 2011; Calvete et al. 2013; Stewart et al. 2004).

There may be two explanations for these contradictory findings—other than differences in design, samples and measurements. The relationship between a negative cognitive style and depressive symptoms could be explained by another variable, or the relationship is spurious due to a confounding variable and disappears after controlling for the confounding variable. Previous longitudinal research has revealed that time-varying factors partly account for the variance of both depressive symptoms and a negative cognitive style (LaGrange et al. 2011).

Negative life events could be such a time-varying variable and might act as a confounding variable and thus alter the link between depressive symptoms and a negative cognitive style. Negative life events are found to be associated with higher levels of depressive symptoms (Hammen 2005) and a more negative cognitive style (Calvete 2011; Hankin 2008; Hankin et al. 2001). Although cognitive theories hypothesize that a negative cognitive style is a cause for depressive symptoms, with negative life events acting as a moderator (Abramson et al. 1989; Beck et al. 1979), a meta-analytic review on this topic, that included studies with two assessment waves (e.g., Abela and Payne 2003; Abela and Seligman 2000; Hankin et al. 2001; Nolen-Hoeksema et al. 1992; Southall and Roberts 2002), showed that the overall interaction effect of a negative cognitive style and negative life events on depressive symptoms was small (partial correlation of 0.22; Lakdawalla et al. 2007). In addition, recent empirical research showed that the interaction between a negative cognitive style and negative life events did not predict depression until late adolescence (Braet et al. 2013). Moreover, these cognitive theories do not explain bidirectional relationships that have been found between negative life events, negative cognitive style and depressive symptoms. A recent study revealed that each variable predicted the others: depressive symptoms predicted a negative cognitive style and negative life events, a negative cognitive style predicted depressive symptoms and negative life events, and negative life events predicted depressive symptoms and a negative cognitive style (Calvete et al. 2013).

As negative life events have thus been found to predict both a negative cognitive style and depressive symptoms (Barrocas and Hankin 2011), the prospective relationships between depressive symptoms and a negative cognitive style may be altered by the experienced negative life events. As we were specifically interested in the prospective relationship between a negative cognitive style and depressive symptoms, we tested whether this relationship remained meaningful when we included or excluded the role of negative life events in the models.

Life events are divided into independent and dependent life events. Independent life events occur without the influence of the person him- or herself (e.g., divorce of parents), while dependent life events refer to events to which the person has contributed, and include most interpersonal events (e.g., conflicts; Hammen 2005). Depressed individuals experience more dependent negative events than independent negative events, and dependent events are highly predictive of depressive symptoms and episodes in adults (Kendler et al. 1999) and adolescents (Auerbach et al. 2011; Shih et al. 2006). Moreover, dependent life events have also been found to be the result of a depressive episode both in a community sample (Patton et al. 2003) and clinically depressed children and adolescents (Rudolph and Hammen 1999; Rudolph et al. 2000). Because dependent life events are most strongly related to depression, we included these in our study.

## The Current Study

As cognitive-behavioral depression prevention programs in community samples do not show the effects as hoped, teaching adolescents skills to change a negative cognitive style to prevent them from developing depressive symptoms may not be an effective approach. One explanation could be that a negative cognitive style does not precede the depressive symptoms in early adolescents. Although cross-sectional studies established that a negative cognitive style and depressive symptoms are related, few empirical studies used a longitudinal design to reveal the temporal ordering of those associations in adolescence. Empirical studies on the bidirectional relationship between depressive symptoms and a negative cognitive style have shown mixed results. Moreover, these studies have not always included dependent negative life events, while these events could alter or confound the relationship between a negative cognitive style and depressive symptoms. The present study contributes to the field by examining bidirectional relationships between a negative cognitive style and depressive symptoms and the role of dependent negative life events regarding these relationships. We used data that covered four assessments over an 18-month period and that were collected as part of an effectiveness study of a depression prevention program (Kindt et al. 2014).

The associations between a negative cognitive style and depressive symptoms were first analyzed without taking into account the potential role of dependent negative life events, and subsequently we considered dependent negative life events in the analytic models by using two different strategies. First, dependent negative life events were added as a variable to the basic model next to depressive symptoms and a negative cognitive style to analyze temporal relationships between those three variables. This way, we also examined the robustness of the relationship between a negative cognitive style and depressive symptoms when including the temporal influence of dependent negative life events. Second, we added dependent negative life events as a time-varying covariate to the basic model, to control for their confounding impact on the relationship between a negative cognitive style and depressive symptoms. We hypothesized that depressive symptoms are both preceding and following a negative cognitive style when dependent negative life events were not included. Further, with respect to the two additional analytic models, we hypothesized that the bidirectional associations between depressive symptoms and a negative cognitive style are less prominent when considering the role of dependent negative life events.

## Methods

### Participants

A total of 1343 adolescents participated in a randomized controlled study on the effectiveness of a depression prevention program. Both participants in the control and intervention condition were included in the analysis, whereby 49.7 % of the adolescents received a depression prevention program. All participants were recruited from the 1st and 2nd grade of secondary education in The Netherlands (7th and 8th grade in USA) and the mean age was 13.4 years (SD = 0.77). Of the total group, 52.3 % were girls and 52.3 % were immigrants (i.e., they themselves or one of their parents were not born in the Netherlands). Because adolescents with parents with psychopathology have an increased risk to develop a depressive disorder (Avenevoli and Merikangas 2006), we asked adolescents whether their parents had been treated by a psychiatrist. Parental psychopathology was reported by 5.9 % of the adolescents, and was controlled for in the analyses.

### Procedure

Schools with at least 30 % of their pupils living in low-income areas in the Netherlands were approached to participate in the study and eleven schools were willing to participate with 57 classes. Parents of the pupils were notified and had the option to refuse data collection from their child by passive consent. Data were collected at four time points with 6-month intervals. The adolescents completed the online self-report questionnaires on school grounds during class time at T1 (December 2011), T2 (June 2012), T3 (December 2012) and T4 (June 2013). The intervention was between T1 and T2. The retention rates at T1 and T2 were high (T1: 93.7 % and T2: 85.8 %) and drop out was primarily due to sickness or absence. Between T2 and T3, 12.3 % dropped out due to change of schools. The participation rates at T3 and T4 were respectively 72.5 and 74.5 %. A flowchart can be found in another study (Kindt et al. 2014). Attrition at T4 was analyzed with logistic regression analyses in which dropout was the dependent variable (0 = dropout, 1 = in the study), and gender, condition, depressive symptoms at T1, dependent life events at T1, and negative cognitive style at T1 were the predictors. Adolescents lost to follow-up were more likely to be boys (OR 1.58, 95 % CI 1.21–2.06, *p* < 0.01), and to have higher depressive symptoms at baseline (OR 0.96, 95 % CI 0.94–0.99, *p* < 0.01). Condition, dependent life events and negative cognitive style did not predict dropout. The local Ethical Committee of the university approved the study (ECG13042011).

### Measurements

#### Depressive Symptoms

Depressive symptoms were assessed with the Children’s Depression Inventory (CDI; Kovacs 1985), which consists of 27 items comprising affective, cognitive and behavioral symptoms of depressive symptoms. The item assessing suicidal ideations was not included in the current study to improve the collaboration with the schools and parents. Per item, adolescents selected one of the three statements that applied best to them in reference to the last 2 weeks. Scores are rated on a three-point scale from 0 to 2 [e.g., “I feel like crying once in a while” (0), “I feel like crying on most days” (1), “I feel like crying every day” (2)]. The total score ranged from zero to 52, with a higher score indicating higher depressive symptoms. The CDI has shown good internal consistency and validity (Evers et al. 2000). The alpha coefficients in the current study were 0.85, 0.88, 0.90 and 0.90 at T1–T4 respectively.

#### Negative Cognitive Style

Negative cognitive style was assessed with the Adolescent Cognitive Style Questionnaire (ACSQ; Hankin and Abramson 2002), which is a recommended assessment tool for measuring cognitive vulnerability because of its good psychometric properties (Lakdawalla et al. 2007). The questionnaire presents hypothetical negative life event scenarios. Based on the cultural fit for adolescents from our country, 7 of the 12 negative scenarios of the original questionnaire were selected and translated to Dutch by native Dutch health care professionals. To check the content of the Dutch version it was translated backwards by native English professionals. Examples of selected scenarios are “you take a test and get a bad grade” and “you want a boyfriend/girlfriend but you don’t have one”. The participants were presented with these hypothetical negative scenarios and were asked to rate the degree to which the cause of the event is internal (i.e., caused by oneself), stable (i.e., the cause remains over time), and global (i.e., the cause will also influence other situations). Next, the participants rated the likelihood of future negative consequences due to the event, and rated the extent to which they believe that what happened shows the person’s self is flawed. Response scales ranged from 1 to 7, with a higher score representing a more negative cognitive style [e.g., from “the event was caused by something else” (1), to “the event was completely caused by myself” (7)]. We used the total score of the questionnaire, as has been done in previous research (e.g., Calvete et al. 2013). The questionnaire displayed excellent internal consistency reliability and good test-retest reliability (Hankin and Abramson 2002). The alpha coefficients in the current study were 0.95, 0.97, 0.98 and 0.98 at T1–T4 respectively.

#### Dependent Negative Life Events

Dependent negative life events were assessed with the Adolescent Life Events Questionnaire-Revised (ALEQ-R; Auerbach et al. 2011). The questionnaire is derived from the ALEQ, which has good validity and reliability (Hankin and Abramson 2002). The ALEQ-R comprises 29 items of negative life events that are dependent and interpersonal in nature. Examples of items are “you got into a fight or argument with your girlfriend/boyfriend” and “you got in trouble with the teacher or principal”. Participants are asked to rate, on a five-point Likert scale ranging from never (0) to always (4), how often these dependent negative life events occurred during the past 3 months. Total scores range from 0 to 116 with higher scores reflecting more negative life events. Cronbach’s alphas in the current study were 0.89, 0.94, 0.94 and 0.96 at T1 to T4 respectively.

### Statistical Analyses

Descriptive statistics were obtained and Pearson correlations were computed for all variables included in this study. We tested baseline differences in depressive symptoms, negative cognitive style and dependent negative life events for age, gender, parental psychopathology, and immigration status using independent-samples *t* tests, which provided us with information about which variables were to be included as covariates in the model.

We applied structural equation modeling using the software package Mplus 5.1 (Muthén and Muthén 1998–2007) to examine longitudinal relationships between depressive symptoms and a negative cognitive style. To make optimal use of the data, we handled missing values with the full-information maximum likelihood (FIML) approach in Mplus. In the basic cross-lagged model, we examined a path diagram that included the paths between the four adjacent measurements of depressive symptoms and negative cognitive style (i.e., autoregressive paths), the associations between measurements of depressive symptoms and negative cognitive style per time point (i.e., cross-sectional associations), and the paths between the two distinct constructs across adjacent time points (i.e., cross-lagged paths). The cross-lagged paths (e.g., between depressive symptoms T1 and negative cognitive style T2) were estimated to examine the effects over time.

In the second model, we added dependent negative life events as a variable next to depressive symptoms and negative cognitive style to analyze the temporal relationships between these three variables. In the third model we added dependent life events as a time-varying covariate to the basic model, by which we controlled for the cross-sectional role of dependent negative life events at each time-point. The parameters in the models were estimated with maximum likelihood estimation with robust standard errors (MLR) to accommodate for skewness of the data. To control for the possible impact of nestedness of the data within classes, we used the type is complex option in Mplus. The root mean square (RMSEA, preferably .05 or lower, and satisfactory between .05 and .08), and the comparative fit index (CFI, preferably .95 or higher) served as model fit indices (Hu and Bentler 1999; Iacobucci 2010).

We used multi-group analyses in Mplus for all three models to test whether the observed cross-lagged associations were moderated by intervention condition and gender. We used the Chi square difference test and compared the unconstrained model (no constraints on cross-lagged paths) with a constrained model in which cross-lagged paths were constrained to be equal. We first constrained all cross-lagged paths simultaneously. A difference in the relative strength of the associations (i.e., moderation effect) would be present for the overall models when a significant Chi square test (*p* value <.05) would be found. In that case, each cross-lagged path would be tested separately to examine which specific paths would be moderated by condition or gender.

## Results

### Descriptives

Table 1 displays the means and standard deviations for all measurements. The sample average of depressive symptoms ranged from 8.5 to 9.5 across assessments, which is in the normative range (Timbremont et al. 2004). At baseline, 7.2 % of the adolescents scored within the clinical range (CDI >19). Table 1 also depicts the correlations among all assessment points of depressive symptoms, negative cognitive style and dependent negative life events. All correlation coefficients were significant and positive, indicating that higher depressive symptoms were associated with a more negative cognitive style and associated with more dependent negative life events.

**Table 1:** Pearson correlations among depressive symptoms, negative cognitive style and life events, and means and standard deviations

Measure	1	2	3	4	5	6	7	8	9	10	11	12
1. Depressive symptoms t1												
2. Depressive symptoms t2	.58											
3. Depressive symptoms t3	.53	.57										
4. Depressive symptoms t4	.41	.45	.57									
5. Negative cognitive style t1	.44	.25	.25	.16								
6. Negative cognitive style t2	.26	.43	.31	.27	.35							
7. Negative cognitive style t3	.25	.29	.46	.28	.36	.50						
8. Negative cognitive style t4	.26	.25	.28	.44	.31	.40	.45					
9. Life events t1	.59	.35	.31	.28	.38	.25	.20	.21				
10. Life events t2	.41	.67	.47	.34	.25	.47	.30	.23	.46			
11. Life events t3	.35	.42	.62	.35	.26	.38	.48	.29	.38	.54		
12. Life events t4	.28	.34	.37	.56	.19	.28	.28	.43	.34	.43	.46	
Mean	8.55	9.47	9.64	9.62	1.88	2.01	2.07	2.10	12.74	15.77	16.02	16.40
SD	6.51	7.72	8.19	8.48	.93	1.07	1.19	1.18	11.12	15.69	15.97	17.47

All correlations are significant at the 0.01 level (two-tailed)

We conducted *t* tests on the baseline scores of depressive symptoms, negative cognitive style and dependent negative life events to see whether they differed with regard to age, gender, parental psychopathology and immigration status. The baseline depressive symptoms were higher for girls than for boys (9.00 vs. 8.07, *p* < .05) and for adolescents whose parents were reported to have psychopathology compared to adolescents whose parents were not reported to have psychopathology (11.89 vs. 8.34, *p* < .001). Baseline depressive symptoms did not differ with regard to immigration status or age. Baseline negative cognitive style was higher for adolescents with parents with psychopathology compared to those with parents without psychopathology (2.16 vs. 1.86, *p* < .01), and for native Dutch adolescents compared to immigrants (1.96 vs. 1.80, *p* < .01), but did not differ for age and gender. Baseline dependent negative life events were higher for girls than boys (13.95 vs. 11.42, *p* < .001), and higher for adolescents with parents with psychopathology compared to those with parents without psychopathology (20.6 vs. 12.24, *p* < .001), but did not differ with regard to age and immigration status. No baseline differences on depressive symptoms, a negative cognitive style and dependent negative life events were found for age. Yet, based on significant Pearson correlations of age with baseline depressive symptoms and dependent negative life events, we decided to control for age next to gender, parental psychopathology, immigration status and condition.

### Relationships Between Depressive Symptoms and a Negative Cognitive Style Over Time

**Fig. 1:**
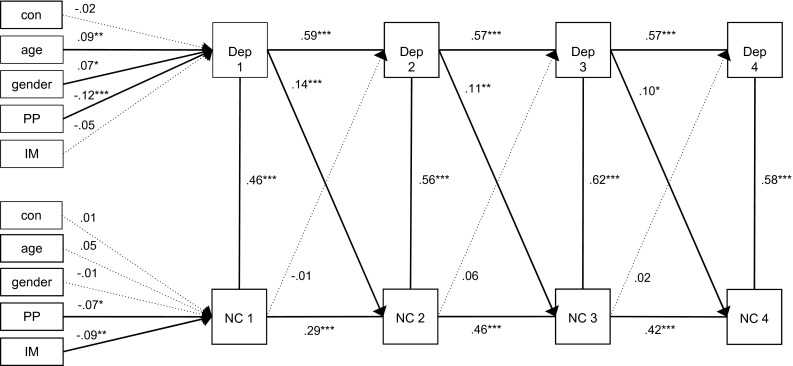
Cross-lagged model among depressive symptoms and a negative cognitive style. *Note* The model provides standardized parameters. *p < .05; **p < .01; ***p < .001. *Dep* depressive symptoms, *NC* negative cognitive style, *PP* parental psychopathology, *IM* immigration status, *Con* study condition. RMSEA .052, CFI .90, Chi square = 1562.5 (*df* = 68, *p* = .000)

Figure 1 displays the results of the basic cross-lagged model among depressive symptoms and a negative cognitive style. The model demonstrated reasonable fit to the data, CFI = .90 and RMSEA = .056. The notion that the CFI is a little lower than the recommended threshold of .95 might indicate that the model is not optimally specified. The autoregressive path coefficients were significant indicating that depressive symptoms and negative cognitive style had a significant stability over time. Cross-sectional associations between depressive symptoms and negative cognitive style were significant and positive. Most importantly, across all four measurements, we found significant and positive associations for the cross-lagged paths from depressive symptoms to negative cognitive style, indicating that higher depressive symptoms predicted an increase in negative cognitive style 6 months later. Negative cognitive style did not predict depressive symptoms 6 months later. Multi-group analyses revealed no differences in cross-lagged paths for intervention condition [∆χ^2^(6) = 9.0, *p* > 0.05], but did show differences for gender [∆χ^2^(6) = 20.7, *p* = 0.002]. Additional multi-group analyses per cross-lagged path for gender revealed that only the cross-lagged path from depressive symptoms at T3 to negative cognitive style at T4 differed [∆χ^2^(1) = 794.4, *p* = 0.000], showing standardized beta’s of −.02 for boys and .22 for girls. Higher depressive symptoms at T3 predicted a higher negative cognitive style at T4 in girls, but not in boys.

### Relationships Between Depressive Symptoms, a Negative Cognitive Style and Dependent Negative Life Events Over Time

**Fig. 2:**
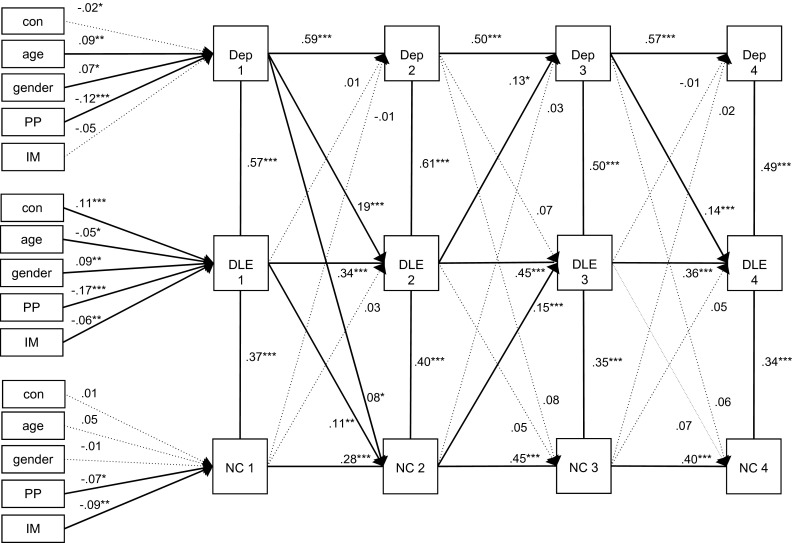
Cross-lagged model among depressive symptoms, a negative cognitive style and dependent negative life events. *Note* The model provides standardized parameters. *p < .05; **p < .01; ***p < .001. *Dep* depressive symptoms, *DLE* dependent negative life events, *NC* negative cognitive style, *PP* parental psychopathology, *IM* immigration status, *Con* study condition. RMSEA = .048, CFI = .94, Chi square = 292.4 (*df* = 72, *p* = .000)

Dependent negative life events were added to the model and showed positive associations with depressive symptoms and negative cognitive style at all time points, as is shown in Fig. 2. The autoregressive paths and cross-sectional paths were all positive and significant. Compared to the basic model in which the variable dependent negative life events was not included, only the cross-lagged path from depressive symptoms to negative cognitive style between T1 and T2 remained significant, while the cross-lagged paths between T2–T3 and T3–T4 disappeared. Negative cognitive style did not predict depressive symptoms at any time point. Significant cross-lagged paths were revealed between depressive symptoms and dependent negative life events. Depressive symptoms at T1 predicted negative life events at T2, with the latter predicting depressive symptoms at T3. Finally, depressive symptoms at T3 predicted negative life events again at T4. Two significant cross-lagged paths revealed between a negative cognitive style and dependent negative life events: dependent negative life events at T1 predicted negative cognitive style at T2, and the latter predicted dependent negative life events at T3. The findings do not indicate a mediating role for dependent negative life events in the relationship between a negative cognitive style and depressive symptoms. Multi-group analyses showed that gender and condition did not have moderating effects.

### Relationships Between Depressive Symptoms and a Negative Cognitive Style Over Time with Dependent Negative Life Events as a Time-Variate Covariate

**Fig. 3:**
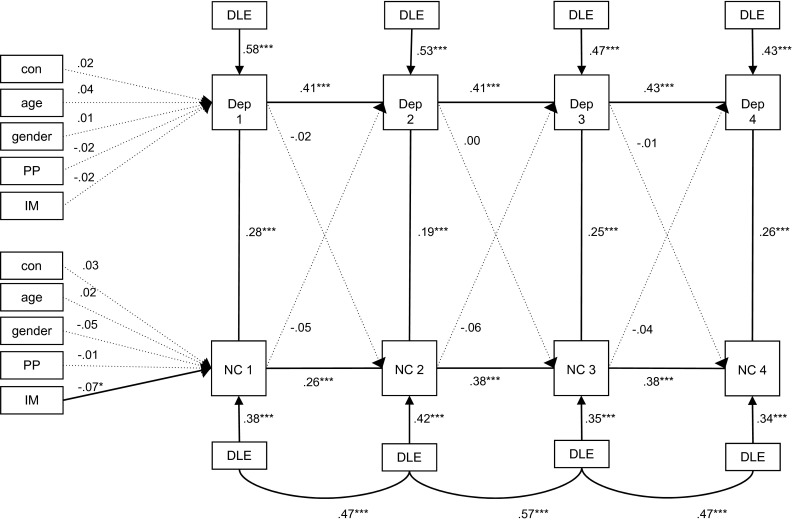
Cross-lagged model among depressive symptoms and a negative cognitive style with dependent negative life events as a time-varying covariate. *Note* The model provides standardized parameters. *p < .05; **p < .01; ***p < .001. *Dep* depressive symptoms, *NC* negative cognitive style, *PP* parental psychopathology, *IM* immigration status, *Con* study condition, *DLE* dependent negative life events. RMSEA = .052, CFI = .91, Chi square = 386.406 (*df* = 84, *p* = .000)

We created the third model by adding dependent negative life events as a time-varying covariate to the basic model (see Fig. 3). Dependent negative life events showed significant and positive associations with depressive symptoms and negative cognitive style at all time points. Of the variables that were controlled for at baseline, none remained associated with baseline depressive symptoms, and only immigration status was still associated with baseline negative cognitive style showing that native Dutch adolescents had a more negative cognitive style. The autoregressive path coefficients remained significant, although in the model with dependent negative life events as a time-variate covariate they seem to have lower values compared to the basic cross-lagged model in which dependent negative life events were not included (for depressive symptoms ranging from .41 to .44 compared to ranging from .57 to .59 in the basic model; and for negative cognitive style ranging from .26 to .38 compared to ranging from .30 to .46 in the basic model). The cross-sectional associations between depressive symptoms and a negative cognitive style remained significant, although the standardized parameters decreased from ranging between .37 and .43 in the basic cross-lagged model to ranging between .19 and .28 in the model in which dependent negative life events were addes as time-varying covariates. Most importantly, none of the cross-lagged paths remained significant, indicating that when we controlled for dependent negative life events, depressive symptoms were not related with a negative cognitive style over time, or vice versa. Multi-group analyses showed that gender and condition did not have moderating effects.

## Discussion

Because high levels of depressive symptoms proceed the onset of a depressive disorder, which is the most serious mental health problem in adolescence (Birmaher et al. 2002), and depressive symptoms in adolescence are known to be predictive for major depressive episodes in adulthood (Pine et al. 1999), prevention of depressive symptoms in adolescence is of importance to improve mental health care. So far, the efforts have mainly been focused on cognitive-therapy based depression prevention in early adolescence, as from late childhood into middle adolescence, individuals develop a more stable cognitive style and are capable of abstract thinking (Cole et al. 2008; Steinberg 2005). Yet, cognitive-therapy based depression prevention programs in adolescents in a community setting have only showed small effects (Merry et al. 2012). Because research showed mixed findings on the relationships between depressive symptoms and a negative cognitive style in adolescents (Calvete 2011; Calvete et al. 2013; Hankin et al. 2001; Johnson and Miller 1990; Kindt et al. 2015; LaGrange et al. 2011; McCarty et al. 2007; Stewart et al. 2004; Timbremont and Braet 2006), we assumed that these programs may not have shown larger effects because the targeted negative cognitive style does not precede depressive symptoms in early adolescence. Hence, the first aim of the study was to test the bidirectional relationships between depressive symptoms and a negative cognitive style. Moreover, because of the strong links of both depressive symptoms and a negative cognitive style with dependent negative life events (Abramson et al. 1989; Auerbach et al. 2011; Beck et al. 1979), we also aimed to test whether the relationship between a negative cognitive style and depressive symptoms remained meaningful when considering dependent negative life events.

Our findings consistently demonstrated that, in a sample of young adolescents with depressive symptoms in the normative range, a negative cognitive style did not predict depressive symptoms. In addition, when we did not consider the role of dependent negative life events in the analyses, the opposite direction was revealed: depressive symptoms predicted a negative cognitive style. Yet, when dependent negative life events were added as a variable next to depressive symptoms and negative cognitive style, the prospective relationship between depressive symptoms and a negative cognitive style did not maintain consistently over time. Furthermore, the prospective association of depressive symptoms on negative cognitive style disappeared when we controlled for the presence of dependent negative life events. These models including dependent negative life events were invariant for gender and intervention condition. Our findings indicate that (a) a negative cognitive style does not seem directly predictive of depressive symptoms, and (b) a longitudinal relationship between depressive symptoms and a negative cognitive style is hard to interpret when dependent negative life events are not taken into account. As a negative cognitive style did not precede and predict depressive symptoms, we may need to revisit the idea of preventing depression in a community sample of early adolescents by changing a negative cognitive style.

Previous longitudinal research among community samples of adolescents that included three or more measurements of depressive symptoms and a negative cognitive style, but did not include dependent negative life events, showed contradicting findings (LaGrange et al. 2011; Kindt et al. 2015). One study found depressive symptoms to be predictive of a negative cognitive style (LaGrange et al. 2011), while another found a negative cognitive style to be predictive of depressive symptoms (Kindt et al. 2015). The variations in findings between these studies and the current study might partly be explained by differences in questionnaires used for assessing depression and a negative cognitive style, or participant characteristics such as the age range. As important, both studies (LaGrange et al. 2011; Kindt et al. 2015) differed from the current study because dependent negative life events were not measured and could thus not be added to the analyses. When dependent negative life events would have been controlled for in those studies, as we did in the current study, the predictive relationships between depressive symptoms and a negative cognitive style may also have disappeared.

Previous studies that are most similar to ours also used cross-lagged panel designs, the same questionnaire to measure negative cognitive style, and a questionnaire for dependent negative life events, however, they did not control for the dependent life events (Calvete 2011; Calvete et al. 2013). With regard to the relationship between depressive symptoms and negative cognitive style, they found reciprocal associations (Calvete et al. 2013) or a more mixed pattern depending on which subscale of the cognitive style questionnaire was analyzed (Calvete 2011). Why different results were found could be due to stronger associations in the current study between dependent negative life events on the one hand, and depressive symptoms and negative cognitive style on the other hand compared to Calvete and colleagues’ studies. Between depressive symptoms and dependent negative life events, the Pearson correlations ranged between .56 and .67 in our study versus between .30 and .40 (Calvete 2011), and between .29 and .36 (Calvete et al. 2013). Between negative cognitive style and dependent negative life events the Pearson correlations ranged between .38 and .48 in the current study, and ranged between .10 and .27 and between .22 and .28 in Calvete’s studies (respectively Calvete 2011; Calvete et al. 2013). Note that differences can only be concluded with precaution, since the Pearson correlations were based on different sample sizes and different questionnaires for depressive symptoms and dependent negative life events.

The different associations may also reflect different sample characteristics such as cultural differences between Spain (Calvete 2011; Calvete et al. 2013) and The Netherlands, or between a mainly native sample (Calvete 2011; Calvete et al. 2013) versus a sample with a high proportion of immigrants, which may be more vulnerable to develop depressive symptoms (Siegel et al. 1998). Although we included adolescents from schools with a high proportion of pupils living in low-income areas who may be at higher risk to develop depressive symptoms during adolescence, we have labeled our sample as a community sample as it shows normative depressive symptoms that are similar to a Dutch universal sample (Tak et al. 2014).

Nevertheless, because of the higher cross-sectional correlations between depressive symptoms, negative cognitive style and dependent life events in the current study, controlling for the dependent negative life events had a higher restrictive role on the association between depressive symptoms and a negative cognitive style than it would have had in the studies of Calvete and colleagues (Calvete 2011; Calvete et al. 2013). As such, the question remains whether controlling for dependent negative life events in those studies would also have led to the same conclusions as presented in the current study, that is, that depressive symptoms and a negative cognitive style are prospectively unrelated when controlled for dependent negative life events.

### Implications

If replicated, this study has important implications for the perspective of depression prevention programs in samples of adolescents with normative depressive symptoms. Most depression prevention programs for community samples of young adolescents are based on principles from cognitive behavioral therapy and target a negative cognitive style (e.g., Gillham et al. 2007; Kindt et al. 2014; Pössel et al. 2004; Stice et al. 2009). Even though the impact of those programs is encouraging, it is not considered satisfying (Merry et al. 2012). Based on the present findings, we do not have evidence that a depression prevention program will be able to prevent depressive symptoms by changing a negative cognitive style, because negative cognitive style did not predict depressive symptoms over time. Yet, the merit of the impact of dependent negative life events on the development of both a negative cognitive style and depressive symptoms should be acknowledged. The occurrence of dependent negative life events was associated with depressive symptoms over time, hence, depression prevention programs aiming a reduction of dependent negative life events might be more effective in reducing depression levels than programs aiming a change in a negative cognitive style. As dependent negative life events cover mostly interpersonal events to which the person has contributed (Hammen 2005), prevention programs could focus on reducing dependent negative life events by social skills training to reduce conflicts with peers or parents. Also, anti-bullying projects, for a better school atmosphere, or programs teaching parents skills how to positively interact with their adolescent might reduce dependent negative life events in youth. Yet, many existing depression prevention programs already involve social problem solving skills (e.g., Gillham et al. 2007; Pössel et al. 2004), and a review showed that the specific content of depression prevention programs (e.g., reducing negative cognitions or focussing on problem-solving) was unrelated to the effect-sizes (Stice et al. 2009). Additional research on the depression prevention programs that reduce dependent negative life events could shed light on this field. As the design of the current study does not allow to draw conclusions on the causal interpretation of the relationship between dependent negative life events and depressive symptoms, additional research is first needed to reveal whether dependent negative life events cause an increase of depressive symptoms in young adolescents from a community sample.

### Limitations

Several limitations provide opportunities for further research. First, we only used self-report questionnaires. Although self-reports receive criticism because of potential biases, it is a widely used strategy that is well suited for assessing human cognitive and emotional states when it fits in the theoretical context in which it is used (Haeffel and Howard 2010). Self-reports are a more accurate approach for assessing emotional states and cognitions compared to the use of parents or teachers as source for information (DiBartolo et al. 1998) and specifically for life events, findings suggest that self-reports and interviews may be equally viable methods (Wagner et al. 2006). Moreover, the use of self-reports enhanced comparison with previous research on depressive symptoms and a negative cognitive style that is also based on self-report measurements. Specifically for measuring parental psychopathology, a multi-informant strategy would have been more reliable and valid than questioning the adolescent whether their parents were ever treated by a psychiatrist. Assessing parental psychopathology the way we did probably excluded parents who had psychopathology but did not receive treatment, and also treated parents whose children did not know they received treatment.

Concerning the dependent negative life events measurements, we formulated three limitations. First, we only measured the frequency of dependent negative life events and did not include the perceived severity or impact of the events, which may have deflated the relationship between dependent negative life events and depressive symptoms (Cohen et al. 1983). Second, we found strong cross-sectional correlations between the variables dependent negative life events, depressive symptoms and a negative cognitive style. Adolescents may have reported dependent negative life events in line with their negative cognitive style and depressive symptoms, resulting in an inflated internal consistency of the dependent life events measure and an over-report of these dependent negative life events. More precisely, the dependent negative life events may be over-reported by those suffering from a negative cognitive style, as those individuals may excessively pay attention to negative aspects of a situation and thereby casting the whole situation in a negative context (Beck et al. 1979). In accordance with that, youth with clinical depressive symptoms are found to have an attentional bias toward negative interpersonal stimuli (Gotlib et al. 2004). Also, previous findings in adult samples suggested that a depressed mood (Shrout et al. 1989) and a negative cognitive style (Simons et al. 1993) influence over-reporting of negative life events on self-report scales. However, depressive symptoms and a negative cognitive style were not found to be associated with over-reporting of events in community children and adolescents compared to interviews (Wagner et al. 2006). Whether over-reporting has happened could be disentangled by the use of multi-informant strategies in future research, such as questionnaires or interviews with parents, peers or teachers. A third limitation is that we did not assess independent life events, which include major life events such as a divorce by parents, illness or death of family members. Although dependent life events are most important for depression research (Auerbach et al. 2011; Shih et al. 2006), additionally testing the effect of independent life events would contribute to the knowledge about the impact of negative life events on the development of depressive symptoms and a negative cognitive style.

Furthermore, although a large proportion of the immigrant adolescents had an ethnical background that is a minority group in the Netherlands (e.g., Moroccan, Turkish), this was not the case for a substantial proportion of the sample. As such, conclusions drawn about the immigrant adolescents cannot be generalized to minority groups in general. At last, the results of this study cannot be generalized to adolescents with clinical levels of depression. As meta-analyses have shown that indicated prevention efforts are more effective than universal prevention programs, even when the same program is used for a different target group (Merry et al. 2012; Stice et al. 2009), it may be that prospective relationships between a negative cognitive style and depressive symptoms are different among adolescents with elevated depressive symptoms.

Future research is encouraged to replicate the current study by following samples of youth over an extended period of time. As it was beyond the scope of the current study, further research could test existing theories such as the stress generation theory (Hammen 1991; Daley et al. 1997), postulating that negative life events are increased by depression, or scar theory (Lewinsohn et al. 1981), postulating that a negative cognitive style is left after a depressive episode.

## Conclusions

Based on cognitive theories that suggest that a negative cognitive style precedes depression, cognitive-theory based depression prevention programs aim to prevent adolescents from developing depressive symptoms by changing a negative cognitive style. As these programs have shown to have only small effects in universal settings, we examined the longitudinal relationships between depressive symptoms and a negative cognitive style, with additionally considering the role of dependent negative life events. Our findings demonstrated that a negative cognitive style did not predict depressive symptoms in young adolescents with depressive symptoms in the normative range. Moreover, this study showed that, when not including dependent negative life events, findings on the relationship between depressive symptoms and a negative cognitive style may show a misleading pattern. Further research should examine whether the findings are replicated. We cautiously conclude that we may need to revise the idea to focus on changing a negative cognitive style in universal depression prevention programs and suggest that more attention could be given to dependent negative events in the lives of adolescents.
